# Regulatory T Cells as Predictors of Clinical Course in Hospitalised COVID-19 Patients

**DOI:** 10.3389/fimmu.2021.789735

**Published:** 2021-12-02

**Authors:** Sara Caldrer, Cristina Mazzi, Milena Bernardi, Marco Prato, Niccolò Ronzoni, Paola Rodari, Andrea Angheben, Chiara Piubelli, Natalia Tiberti

**Affiliations:** ^1^ Department of Infectious – Tropical Diseases and Microbiology, Istituto di Ricovero e Cura a Carattere Scientifico (IRCCS) Sacro Cuore - Don Calabria Hospital, Verona, Italy; ^2^ Centre for Clinical Research, Istituto di Ricovero e Cura a Carattere Scientifico (IRCCS) Sacro Cuore - Don Calabria Hospital, Verona, Italy

**Keywords:** COVID-19, immunophenotype, T cell subtypes, regulatory T cells, disease severity

## Abstract

**Background:**

The host immune response has a prominent role in the progression and outcome of SARS-CoV-2 infection. Lymphopenia has been described as an important feature of SARS-CoV-2 infection and has been associated with severe disease manifestation. Lymphocyte dysregulation and hyper-inflammation have been shown to be associated with a more severe clinical course; however, a T cell subpopulation whose dysfunction correlate with disease progression has yet to be identify.

**Methods:**

We performed an immuno-phenotypic analysis of T cell sub-populations in peripheral blood from patients affected by different severity of COVID-19 (n=60) and undergoing a different clinical evolution. Clinical severity was established based on a modified WHO score considering both ventilation support and respiratory capacity (PaO2/FiO2 ratio). The ability of circulating cells at baseline to predict the probability of clinical aggravation was explored through multivariate regression analyses.

**Results:**

The immuno-phenotypic analysis performed by multi-colour flow cytometry confirmed that patients suffering from severe COVID-19 harboured significantly reduced circulating T cell subsets, especially for CD4^+^ T, Th1, and regulatory T cells. Peripheral T cells also correlated with parameters associated with disease severity, i.e., PaO2/FiO2 ratio and inflammation markers. CD4^+^ T cell subsets showed an important significant association with clinical evolution, with patients presenting markedly decreased regulatory T cells at baseline having a significantly higher risk of aggravation. Importantly, the combination of gender and regulatory T cells allowed distinguishing between improved and worsened patients with an area under the ROC curve (AUC) of 82%.

**Conclusions:**

The present study demonstrates the association between CD4^+^ T cell dysregulation and COVID-19 severity and progression. Our results support the importance of analysing baseline regulatory T cell levels, since they were revealed able to predict the clinical worsening during hospitalization. Regulatory T cells assessment soon after hospital admission could thus allow a better clinical stratification and patient management.

## Introduction

The worldwide emergency of COVID-19 pandemic has led the scientific community to study in depth the host immune response during this acute viral illness since the broad spectrum of disease severity has suggested an important, although unclear, role of post-infection immunity. In this context, the key role of the T-cell mediated immunity has emerged and the SARS-CoV-2 specific T cell response has progressively been delineated (1, 2). Several studies have already reported that some COVID-19 patients present an impaired T cell response (3) and that severe cases are characterised by dysfunctional cellular and humoral immunity (4, 5). Lymphopenia has thus become a hallmark of COVID-19 severe disease (6, 7). Such alterations have also been shown to affect cell differentiation and increased activated T cells have been reported in severe COVID-19 (8–10). Indeed, in severe subjects, the CD4^+^ T cell response showed a functional impairment associated with an increased expression of exhaustion markers, while a predominant activation of CD8^+^ T cells was observed in mild patients (11). The putative association of Th1/Th2 CD4^+^ cells with disease progression has also been investigated (12, 13), but conflicting results have been reported (14). A relation between systemic hyper-inflammation and COVID-19 severity or progression has also been described. Increased circulating levels of IL-6, IL-8 and TNF-α as well as a diminished production of type I IFN by peripheral blood immune cells at admission were in fact reported as independent predictors of disease outcome and proposed as biomarkers to guide treatment choice (15–18). A more in-depth understanding of the functional role of cell-mediated immunity in COVID-19 pathogenesis through T cell evaluation during the acute phase might be found crucial to help in patients’ management and to prevent severe disease. Indeed, numerous efforts are currently ongoing to decipher the contribution of memory T cells to the adaptive response to SARS-CoV-2 (19) and to develop effective vaccines and disease control measures.

Here we performed a retrospective study to investigate the immune dysregulation associated with COVID-19 severity and clinical course. Specifically, we assessed circulating T cell subsets and systemic cytokines during the acute phase of the infection in 60 hospitalised subjects. In addition to the commonly studied CD4^+^ and CD8^+^ T cells, we also targeted CD4^+^ sub-populations including Th1, Th2, Th17 and regulatory T cells (Tregs). Correlation and regression analyses were performed to establish the potential of specific immunological features for the prediction of COVID-19 clinical aggravation during hospitalisation.

## Methods

### Study Population and Sample Collection

Patients diagnosed with COVID-19 and admitted to the COVID-19 ward of the IRCCS Sacro Cuore Don Calabria Hospital between March and April 2020 were consecutively included. Demography, clinical characteristics and laboratory findings upon admission were retrieved from electronic medical records (**Tables 1**, **2**). Patients were classified into three categories of severity based on a modified WHO score (20) to take into account both the type of ventilation administered and the lung function expressed through the Horowitz index (PaO_2_/FiO_2_ ratio - arterial oxygen partial pressure to inspired oxygen fraction). Patients were thus classified as follows: mild (score 4), if no oxygen therapy was administered or PaO_2_/FiO_2_ ≥300; moderate (score 5), if oxygen supplied by mask or nasal prongs, or PaO_2_/FiO_2_ between 150–299; severe (score≥6), if oxygen administered by NIV, high flow or intubation, or PaO_2_/FiO_2_ <150.

**Table 1:** Baseline demographic and clinical characteristics of COVID-19 patients.

	Mild (n = 23)	Moderate (n = 28)	Severe (n = 9)	p-value^a^	Post-test p-value^b^
**Gender (F), n (%)**	12 (42.9%)	13 (46.4%)	1 (11.1%)	ns	
**Age (years), median [range]**	68 [20-94]	77 [43 - 93]	84 [65 - 98]	0.0215	Mi vs Mo.: 0.042 Mi vs S: 0.023
**Delay between blood collection and symptom onset (days), median [range]**	5.5 [1 - 32]	7 [2 - 26]	8 [1 - 17]	ns	
**Fever (yes), n (%)**	11 (47.8%)	10 (35.7%)	4 (44.4%)	ns	
**PaO2/FiO2 ratio, median [IQR]** ^c^	332.5 [314 - 357]	241 [223 - 275]	83 [75 - 108]	0.0001	Mi vs Mo: <0.001 Mo vs S: 0.012 Mi vs S: <0.001
**Oxygen supply, n (%)**				0.004	
**None**	9 (39.1%)	3 (10.7%)	0		
**Low flow**	14 (60.9%)	25 (89.3%)	7 (77.8%)		
**NIV**	0	0	1 (11.1%)		
**Intubation**	0	0	1 (11.1%)		
**Presence of comorbidities (yes), n (%)**	11 (47.8%)	25 (89.3%)	8 (88.9%)	0.003	
**Type of co-morbidities, n (%)**					
**Diabetes**	3 (13%)	8 (28.6%)	2 (22.2%)		
**Cardiovascular diseases**	9 (39.1%)	13 (46.4%)	6 (66.7%)		
**Hypertension**	5 (21.7%)	11 (39.3%)	2 (22.2%)		
**Neoplasm**	1 (4.3%)	1 (3.6%)	2 (22.2%)		
**Respiratory diseases**	3 (13%)	5 (17.9%)	3 (33.3%)		
**Neurodegenerative diseases**	0	1 (3.6%)	0		
**Hormonal and metabolic disorders**	1 (4.3%)	5 (17.9%)	0		
**Obesity**	1 (4.3%)	1 (3.6%)	0		
**Other chronic diseases**	2 (8.7%)	3 (10.7%)	0		
**Ongoing treatment**^d^ **(yes), n (%)**	16 (69.6%)	18 (64.3%)	6 (66.7%)	ns	
**Type of treatment**^e^**, n (%)**					
**None**	7 (30.4%)	10 (35.7%)	3 (33.3%)		
**Hydroxychloroquine**	7 (30.4%)	9 (32.1%)	1 (11.1%)		
**Hydroxychloroquine + Tocilizumab**	1 (4.3%)	0	0		
**Hydroxychloroquine + antiviral**	6 (26.1%)	9 (32.1%)	5 (55.6%)		
**Hydroxychloroquine + antiviral + corticosteroids**	1 (4.3%)	0	0		
**Hydroxychloroquine + corticosteroids**	1 (4.3%)	0	0		
**Clinical course (worsened), n (%)**	7 (30.4%)	8 (28.6%)	8 (88.9%)	0.005	
**Outcome (death), n (%)**	2 (8.7%)	3 (10.7%)	5 (55.6%)	0.008	

a Kruskal-Wallis test for comparison between continuous variables; Chi-squared or Fisher’s exact test for categorical variables.

b Dunn post-test with Bonferroni correction. Mi, mild; Mo, Moderate; S, Severe.

c Missing data for 1 mild patient and 1 moderate patient.

d Ongoing treatment at the time of blood collection.

e Type of ongoing treatment at the time of blood collection. Antivirals included: darunavir/cobicistat, lopinavir/ritonavir.

IQR, interquartile range; Mild, modified WHO score = 4; Moderate, modified WHO score = 5; Severe, modified WHO score≥6.

ns, non significant.

**Table 2:** Principal laboratory findings at baseline.

	Mild (n = 23)	Moderate (n = 28)	Severe (n = 9)	p-value^a^	Post-test p-value^b^
**WBC (10^9^/L), median [IQR]**	6.1 [4.5 - 7.2]	5.65 [3.95 - 8.25]	9.7 [9 - 11]	0.013	Mo vs. S: 0.008 Mi vs. S: 0.011
**Neutrophil (10^9^/L), median [IQR]**	3.7 [2.52 - 5.2]	4.1 [2.35 - 6.5]	9.1 [7.8 - 10.1]	0.001	Mo vs. S: 0.002 Mi vs. S: <0.001
**Neutrophil %, median [IQR]**	66.6 [51.2 - 75.1]	74.9 [64.3 - 79.95]	89.5 [87.1 - 91.6]	<0.001	Mo vs. S: <0.001 Mi vs. S: <0.001
**Lymphocytes (10^9^/L), median [IQR]**	1.3 [1 - 1.8]	0.95 [0.8 - 1.35]	0.5 [0.4 - 0.6]	<0.001	Mo vs. S: 0.01 Mi vs. S: <0.001
**Lymphocytes %, median [IQR]**	22.6 [17.6 - 32.3]	15.35 [11.4 - 26.45]	5.2 [3.9 - 6.5]	<0.001	Mo vs. S: <0.001 Mi vs. S: <0.001
**Monocytes (10^9^/L), median [IQR]**	0.5 [0.3 - 0.7]	0.5 [0.3 - 0.75]	0.4 [0.2 - 0.6]	ns	
**Monocytes %, median [IQR]**	8.7 [5.8 - 10.1]	8.5 [6.95 - 10.95]	3.5 [3.3 - 4]	<0.001	Mo vs. S: <0.001 Mi vs. S: <0.001
**CRP (mg/L), median [IQR]**	47.2 [26.15 - 74.52]	92.9 [21.65 - 145.5]	124.3 [109.6 - 132]	0.015	Mi vs. S: 0.008
**Ferritin (microg/L), median [IQR]**	275.1 [87.6 - 757.5]	398.5 [183 - 722.8]	1110 [100 - 1486]	ns	
**IL-6 (pg/ml), median [IQR]**	17.36 [8.96 - 37.84]	26.24 [8.64 - 67.48]	67.12 [31.24 - 99.66]	0.048	Mi vs. S: 0.023
**Creatinine (µmol/L), median [IQR]**	74 [67 - 117]	74.5 [64 - 90.5]	108 [106 - 119]	0.012	Mo vs. S: 0.005 Mi vs. S: 0.023
**D-Dimer (µg/L), median [IQR]**^c^	835 [487 - 1530]	1038 [496.5 - 2357]	2482 [1241 - 5400]	ns	
**ACE (U/L)**^d^	26.8 [19.7 - 31.5]	25.2 [5.3 - 39.1]	17.6 [17.7 - 25.6]	ns	
**RT-qPCR Ct, median [IQR]** ^e^	25 [21 - 32]	25 [23 - 31]	24 [19 - 30]	ns	
**IgM-S (positive), n (%)**^f^	12 (52.2%)	15 (53.6%)	7 (77.8%)	ns	
**IgG-N (positive), n (%)**^f^	9 (39.1%)	16 (57.1%)	7 (77.8%)	ns	

a Kruskal-Wallis test for comparison between continuous variables.

b Dunn post-test with Bonferroni correction, Mi, mild; Mo, Moderate; S, Severe.

c Missing data for 1 mild patient.

d Missing data for 2 mild patients.

e Missing data for 2 mild patients and 2 moderate patients.

f Missing data for 4 mild patients, 3 moderate patients and 1 severe patient.

IQR, interquartile range; Mild, modified WHO score = 4; Moderate, modified WHO score = 5; Severe, modified WHO score≥6

ns, non significant.

All patients signed written informed consent. The study was conducted according to the guidelines of the Declaration of Helsinki and approved by the Ethical Committee of Verona and Rovigo provinces under protocol no. 63471/2020.

Whole blood and serum samples used for experimental analyses were collected upon admission and stored at -80°C until further use.

### SARS-CoV-2 Molecular and Serological Tests

COVID-19 diagnosis was performed by reverse-transcriptase real time PCR (RT-qPCR) on nasopharyngeal swab according to WHO guidelines (available at https://www.who.int/publications/i/item/diagnostic-testing-for-sars-cov-2) and applying the CDC 2019-nCoV rRT-PCR Diagnostic Panel assay and protocol (available at https://www.fda.gov/media/134922/download) (21). IgG anti-SARS-CoV2 nucleocapsid protein (IgG-N) and IgM anti-SARS-CoV2 spike protein (IgM-S), were measured in patients’ serum using a chemiluminescent microparticle immunoassays (CMIA) (Abbott, Ireland) following manufacturer’s instructions. Results were expressed as assay index, i.e. sample relative light unit (RLU)/calibrator RLU. The assay was considered positive for indices >1.4 or ≥1 for IgG-N and IgM-S, respectively.

### Flow Cytometry Analyses

Flow cytometry analyses were performed on whole blood samples collected in EDTA, aliquoted and stored at -80°C in 10% DMSO (v/v). The list and concentration of mouse anti-human monoclonal antibodies employed (BD Biosciences, San Jose, CA, USA) are reported in **Table S1**. Samples were prepared as recommended elsewhere (22). Briefly, three test tubes containing different combinations of fluorescence-labelled antibodies were used for each patient. One hundred and twenty (120) μL of whole blood were added to each panel-tube, vortexed and incubated at room temperature for 20 minutes in the dark. Erythrocytes were lysed and sample fixed with 1X FACS Lysing solution (BD Bioscience) for 10 minutes in the dark. Samples were washed twice with PBS + 0.5% BSA (w/v) and cell pellet was finally suspended in 200 μL of PBS + 0.5% BSA. Data acquisition was performed using a CytoFlex flow cytometer (Beckman Coulter, Brea, CA, USA) with the CytExpert software v2.3 (Beckman Coulter). The stopping rule was set at 100000 events in the CD45^+^ gate or, when this criterion could not be met due to severe leukopenia, the entire sample was acquired. The number of cells/µL was determined by the instrument based on the volume of sample consumed. The selection of surface markers and the gating strategy employed are reported in **Figure S1**. Data were analysed with the Kaluza software v2.1 (all from Beckman Coulter, Brea, CA, USA).

### Systemic Cytokine Concentration

The serum concentration of IFN-α, IFN-γ, IL-2, IL-4, IL-5, IL-6, IL-9, IL-10, IL-12p70, IL-17A, TNF-α, GM-CSF was measured using the MACSPlex Cytokine 12 kit (Miltenyi Biotec). Samples were prepared as recommended by the manufacturer. Briefly, samples were centrifuged at 10000*g* for 5 minutes at 4°C to remove large debris and the supernatant was then diluted 1:4 with sample buffer provided within the kit. Data were acquired on a CytoFlex flow cytometer (Beckman Coulter) at a 20 µL/min flow rate. Acquisition stopping rule was set at 4000 events in the bead gate or 180µl of acquired sample. Exported data were analysed with Flowlogic software (Inivai Technologies) and the median intensity in APC was used to extrapolate cytokine concentrations. To all samples having concentration values out of range (OOR), an arbitrary value corresponding to half of the lowest measured concentration was assigned.

### Statistical Analyses

Statistical analyses were performed using STATA software v14.0 (StataCorp LP, TX, USA) or SAS EG v7.1 (SAS Institute Inc., NC, USA), and plots generated with GraphPad Prism v8.3.0 (GraphPad Software, CA, USA). Non-parametric tests were applied according to data distribution. Differences in cell or cytokine concentration were assessed using the Mann-Whitney *U* test or the Kruskal-Wallis test followed by Dunn’s post-test and Bonferroni correction for multiple comparisons. The Spearman coefficient was used to evaluate correlations. Significance level was set at p-value <0.05 and all tests were two-tailed. A linear regression model was used to investigate the effect of disease severity on T cell subsets and cytokines, after log transformation of variables. Significant univariable models were then adjusted for the effects of covariates of interest in multivariable linear regression models. The ability of circulating cells and cytokines to predict the probability of clinical aggravation was explored using Firth logistic regression. Significant regressors (p-value from univariable analyses <0.2) were dichotomized using Receiver Operating Characteristic (ROC) curve cut-off analysis and included in multivariable Firth logistic regression models. Cut-offs were defined maximizing the sum of sensitivity and specificity (Youden’s J statistic) (**Table S6**). Areas Under the Curve (AUC) were compared by the DeLong test. Goodness-of-fit measures were estimated for these models.

## Results

### Population Characteristics

The demographic and clinical characteristics of COVID-19 patients classified as mild, moderate or severe are reported in **Table 1**. No differences in gender frequencies were observed across the three groups, while a small, although significant, difference in patients’ age was observed, with severe and moderate patients being older than mild patients. Considering clinical information, the frequency of subjects presenting with fever was comparable between the three groups. Comorbidities were significantly more frequent among patients suffering from severe or moderate COVID-19 (89% for both groups) compared to the mild form (48%), with cardiovascular diseases being the most common co-morbidity. The number of patients receiving treatment was the same across the three groups and the majority of them were receiving either hydroxychloroquine or a combination of hydroxychloroquine and antivirals, as a reflection of standard of care at the time of the study. The frequency of subjects worsening during hospitalisation or having a fatal outcome was significantly higher among severe patients (89% and 56%, respectively) compared to the other groups.

The principal laboratory findings obtained on blood samples collected at the same time as those tested here are reported in **Table 2**. In agreement with already reported data, disease severity was associated with a general leucocytosis and lymphopenia (23). Severe patients presented a raised concentration of inflammatory markers, i.e., C-reactive protein (CRP) and IL-6, while other markers proposed to be related with SARS-CoV-2 infection severity, including ferritin, D-dimer and angiotensin-converting enzyme were not. Blood creatinine was also higher in severe disease compared to milder forms. No differences in the viral load, expressed as RT-qPCR Ct value, were observed between the three groups. The frequency of patients with positive IgM-S and IgG-N antibodies did not differ across the three groups.

### Lymphocyte Subset Frequencies Are Associated With COVID-19 Severity

**Figure 1:**
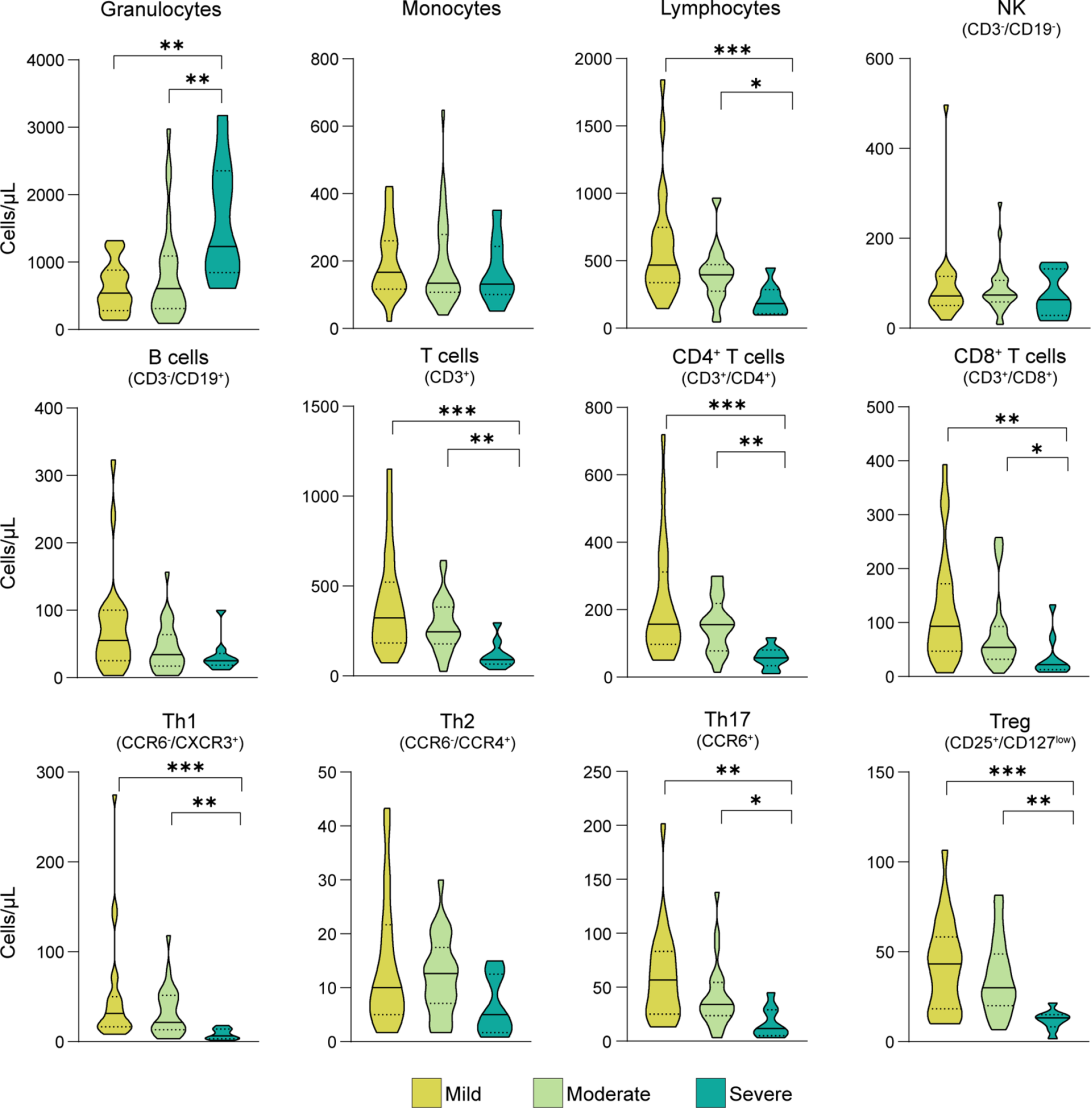
Immunophenotypic analysis in COVID-19 patients classified according to the severity of the disease. Distribution of the absolute number of cells (expressed as cells/µl of blood) across the three groups of COVID-19 patients suffering from different disease severity, i.e. mild or score 4 (n = 23), moderate or score 5 (n = 28), severe or score ≥6 (n = 9), established according to a modified WHO classification (20). Statistical significance, set at p-value <0.05, was assessed using the Kruskal-Wallis test followed by the Dunn’s post-test and Bonferroni correction for multiple comparisons. *p < 0.05; **p < 0.01; ***p < 0.001.

The gating strategy adopted to determine the frequency of leucocytes and lymphocyte subsets is reported in **Figure S1**. A marked reduction in the absolute number of total lymphocytes and T cell subsets (CD4^+^, CD8^+^ T, Th1, Th17 and Treg) was recorded in severe COVID-19 patients compared to mild or moderate subjects, while the number of granulocytes was significantly increased in severe patients (**Figure 1** and **Table S2**).

A multivariate linear regression analysis confirmed the significant association between disease severity and the amount of the different lymphocytes, independently of the effect of age, gender, presence of fever or comorbidities, and ongoing treatment, considered as potential effect modifiers (**Table 3**). In addition, patients’ gender showed a significant association with CD4^+^ T cells, while the presence of fever and comorbidities were associated with Th1 variability.

**Table 3:** Multivariable linear regression analysis.

	Intercept	[95% Confidence Interval]		p-value	Adjusted R^2^
**Granulocytes**					
Age	0.003	-0.005	0.011	0.474	0.09
Gender	0.044	-0.157	0.244	0.665	
Fever	0.062	-0.139	0.263	0.538	
Comorbidities	-0.129	-0.403	0.145	0.349	
Treatment^a^	-0.048	-0.261	0.165	0.653	
Severity					
*Mild*	-0.458	-0.769	-0.147	**0.005**	
*Moderate*	-0.386	-0.669	-0.104	**0.008**	
**Lymphocytes**					
Age	0.000	-0.006	0.006	0.985	0.27
Gender	0.143	-0.004	0.290	0.057	
Fever	-0.060	-0.207	0.088	0.420	
Comorbidities	-0.089	-0.290	0.111	0.376	
Treatment^a^	-0.104	-0.260	0.051	0.185	
Severity					
*Mild*	0.347	0.119	0.574	**0.004**	
*Moderate*	0.220	0.013	0.427	**0.038**	
**T cells (CD3^+^)**					
Age	0.002	-0.005	0.008	0.597	0.30
Gender	0.159	-0.002	0.321	0.053	
Fever	-0.062	-0.224	0.100	0.447	
Comorbidities	-0.100	-0.321	0.121	0.368	
Treatment^a^	-0.079	-0.250	0.093	0.360	
Severity					
*Mild*	0.458	0.207	0.708	**0.001**	
*Moderate*	0.336	0.108	0.563	**0.005**	
**CD4^+^ T cells**					
Age	0.001	-0.006	0.008	0.785	0.36
Gender	0.213	0.050	0.377	**0.011**	
Fever	-0.079	-0.243	0.084	0.334	
Comorbidities	-0.185	-0.408	0.037	0.101	
Treatment^a^	-0.074	-0.247	0.099	0.396	
Severity					
*Mild*	0.401	0.148	0.654	**0.002**	
*Moderate*	0.360	0.130	0.589	**0.003**	
**CD8^+^ T cells**					
Age	0.003	-0.006	0.012	0.471	0.20
Gender	0.147	-0.072	0.367	0.183	
Fever	-0.063	-0.283	0.157	0.570	
Comorbidities	-0.005	-0.305	0.294	0.973	
Treatment^a^	-0.144	-0.377	0.088	0.218	
Severity					
*Mild*	0.560	0.221	0.900	**0.002**	
*Moderate*	0.347	0.038	0.655	**0.029**	
**Th1 (CCR6^-^/CXCR3^+^)**					
Age	0.004	-0.004	0.013	0.309	0.46
Gender	0.273	0.073	0.473	**0.008**	
Fever	-0.242	-0.446	-0.038	**0.021**	
Comorbidities	-0.355	-0.635	-0.075	**0.014**	
Treatment^a^	-0.008	-0.220	0.203	0.937	
Severity					
*Mild*	0.505	0.202	0.808	**0.002**	
*Moderate*	0.391	0.107	0.676	**0.008**	
**Th17 (CCR6^+^)**					
Age	-0.001	-0.009	0.007	0.802	0.29
Gender	0.091	-0.106	0.288	0.357	
Fever	-0.125	-0.326	0.076	0.216	
Comorbidities	-0.199	-0.475	0.077	0.154	
Treatment^a^	-0.070	-0.278	0.139	0.505	
Severity					
*Mild*	0.487	0.189	0.786	**0.002**	
*Moderate*	0.378	0.098	0.659	**0.009**	
**Treg (CD25^+^/CD127^low^)**					
Age	0.003	-0.004	0.009	0.449	0.33
Gender	0.137	-0.024	0.298	0.093	
Fever	-0.087	-0.248	0.074	0.281	
Comorbidities	-0.211	-0.430	0.008	0.059	
Treatment^a^	-0.020	-0.190	0.150	0.815	
Severity					
*Mild*	0.421	0.173	0.670	**0.001**	
*Moderate*	0.426	0.200	0.653	**<0.001**	

Reference categories for categorical variables: Gender = male; Fever = no; Comorbidities = no, Treatment = no, Score = 6.

Significant p-values are reported in bold.

a Treatment administered before blood collection.

Only cell populations significant in the univariable analysis were assessed in the multivariable model.

n = 56 for Th1 and Th17.

### Lymphocyte Subset Counts Correlate With Inflammation Markers and Lung Function but Not With Anti-SARS-CoV-2 Antibodies

**Figure 2:**
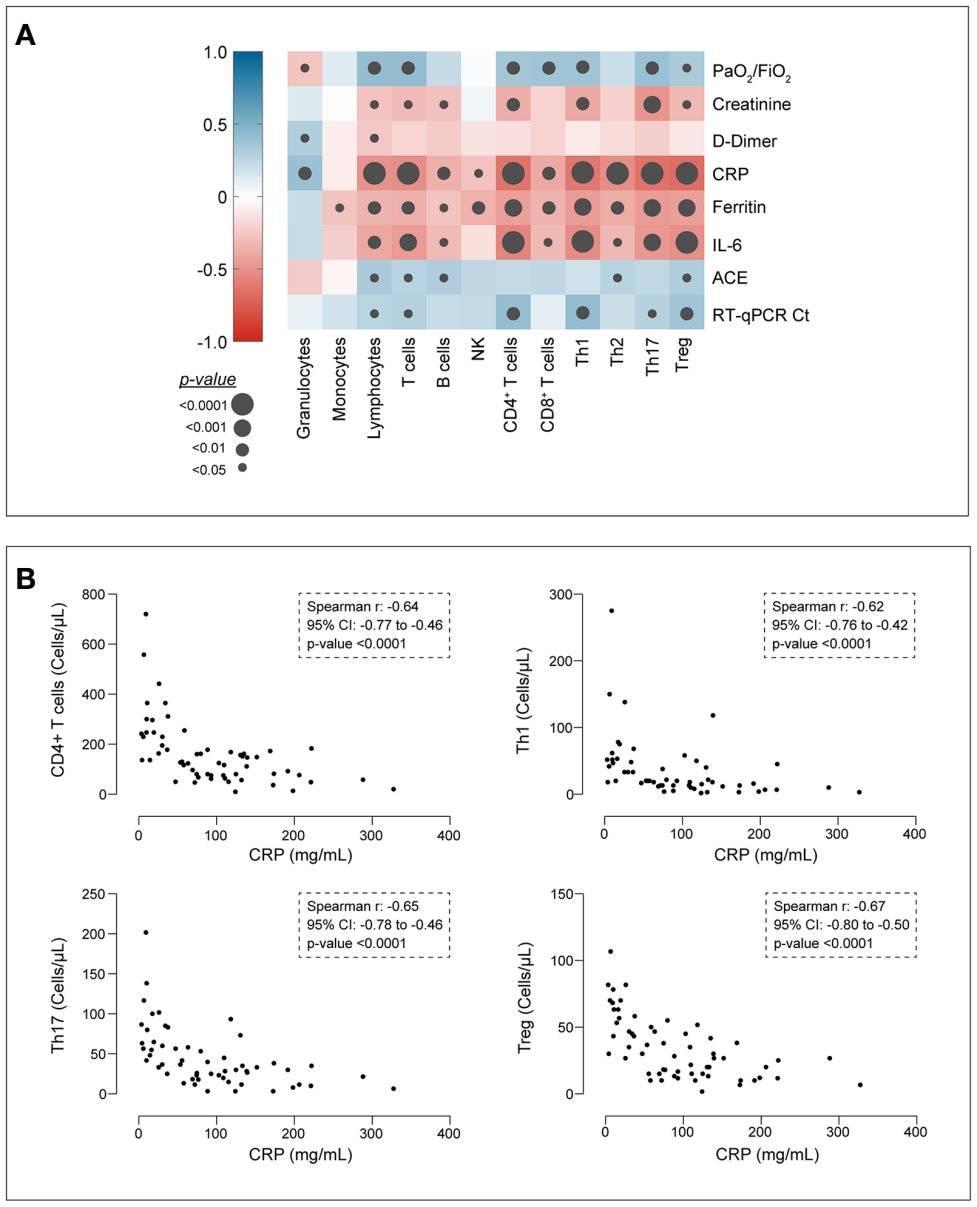
Correlation analysis. **(A)** Correlation matrix assessing the linear correlation between cell types and relevant clinical and biochemical parameters. Colour scale indicates the Spearman *ρ* coefficient; grey dots indicate the significance level. Cells are expressed as cells/µL of blood. PaO_2_/FiO_2_, Horowitz index; creatinine, µmol/L; D-dimer, µg/L; CRP, C-reactive protein, mg/L; ferritin, µg/L; IL-6, interleukin-6 measured at the time of complete blood count, pg/mL (chemiluminescence immunoassay); ACE, angiotensin converting enzyme (U/L); RT-qPCR Ct, number of cycles. **(B)** Detailed scatter plots of correlations displaying Spearman r coefficient > |0.6|.

A correlation matrix was computed to assess whether lymphocyte immuno-phenotype shows a linear relation with clinical and biochemical parameters, independently of disease severity (**Figure 2A**). The Horowitz index showed a significant correlation (p<0.01) with total lymphocytes and CD3^+^ T cells (moderate positive correlation), as well as with CD4^+^ and CD8^+^ T cells, Th1, Th17 and Treg (weak positive correlation), indicating a relation between a reduced lung function and reduced circulating cells of the T compartment. Significant negative correlations were observed between inflammatory markers (CRP, ferritin and IL-6) and the majority of the cell subsets analysed (**Figure 2A**). In particular, the most relevant correlations having a Spearman r coefficient < -0.6 were observed for CRP and CD4^+^ T cells, Th1, Th17 or Treg (**Figure 2B**). Finally, total lymphocytes, T cells, CD4^+^, Th1, Th17 and Tregs showed a significant relation with RT-qPCR Ct values, pointing out that higher viral loads are accompanied by a lower absolute count of T cell subsets (**Figure 2A**). Considering anti-SARS-CoV-2 antibodies, the absolute number of the different cell types analysed did not differ between patients with negative or positive serology, neither for IgM-S nor for IgG-N antibodies (**Table S3A**). Moreover, in patients with positive serology, the cell count did not correlate with IgM-S or IgG-N indices (**Table S3B**).

### The Cytokine Systemic Concentration Is Not Affected by Disease Severity

The systemic levels of selected cytokines were measured in serum samples collected on the same day as blood samples used for the immuno-phenotype analysis. IL-2 and IL-9 were excluded from further analyses as more than 90% of the measured samples had OOR values. The systemic concentration of all measured cytokines did not vary according to COVID-19 severity (**Table S2**) however, when evaluated at the individual level, it was possible to identify within each severity group, clusters of patients displaying raised levels of all or most of the assessed cytokines (**Figure S2**). Among the measured cytokines, only a few correlated significantly with the number of circulating lymphocyte populations, although the strength of the correlation was overall weak (**Table S4**). Only IL-6 and CD4^+^ T cells showed a moderate negative correlation (Spearman rho = -0.54, p-value <0.0001).

### Patients With Decreased Specific T Cell Subsets at Baseline Have Increased Risk of Worsening During Hospitalisation

To evaluate the potential association between our experimental data and patients’ clinical course, our cohort was re-classified according to the progression of patients’ status as improved or worsened compared to the clinical conditions established on the day of sample collection. Clinical aggravation was determined as an increased requirement of oxygen compared to baseline or death during hospitalisation. On this base, we included 37 patients with an improved clinical course and 23 whose conditions worsened. Amongst the latter, 43.5% ultimately died from COVID-19.

**Figure 3:**
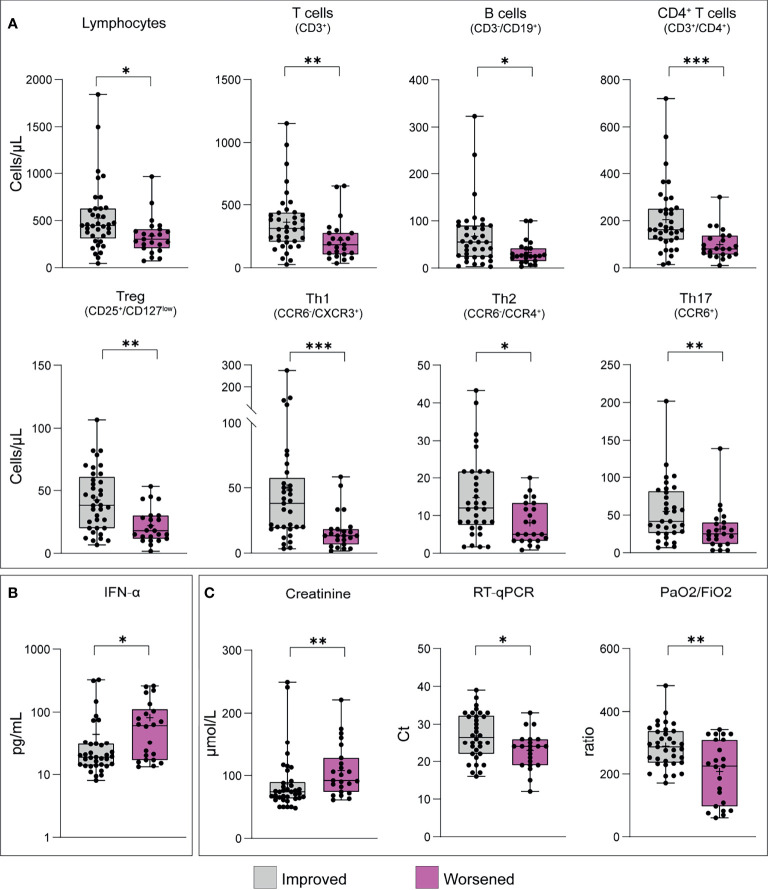
T cell frequencies, cytokine concentrations and laboratory findings in patients classified according to the clinical course during hospitalisation. Patients were classified as improved (n = 37) or worsened (n = 23) as reported in the methods section. **(A)** T cell and T cell subset frequencies; **(B)** cytokine concentration; **(C)** laboratory findings. Only results statistically significant are reported. Statistical significance, set at p-value <0.05, was assessed using the Mann-Whitney U test. *p < 0.05; **p < 0.01; ***p < 0.001. Whiskers represent minimum and maximum values, dots represent individual observations, the + on each box indicates the mean.

Total lymphocytes, B and T cells and T subsets (except for CD8^+^ T cells), were significantly reduced in worsening patients; CD4^+^ T cells and Th1 cells showed the strongest differences between the two groups (p ≤ 0.0006) (**Figure 3A** and **Table S5**). Additionally, worsening patients displayed significantly higher systemic concentrations of IFN-α (p value=0.0175) (**Figure 3B** and **Table S5**). Among the clinical and biochemical parameters already proposed as associated with disease severity, only the viral load, blood creatinine and PaO_2_/FiO_2_ ratio were able to differentiate between the two groups (**Figure 3C** and **Table S5**).

**Figure 4:**
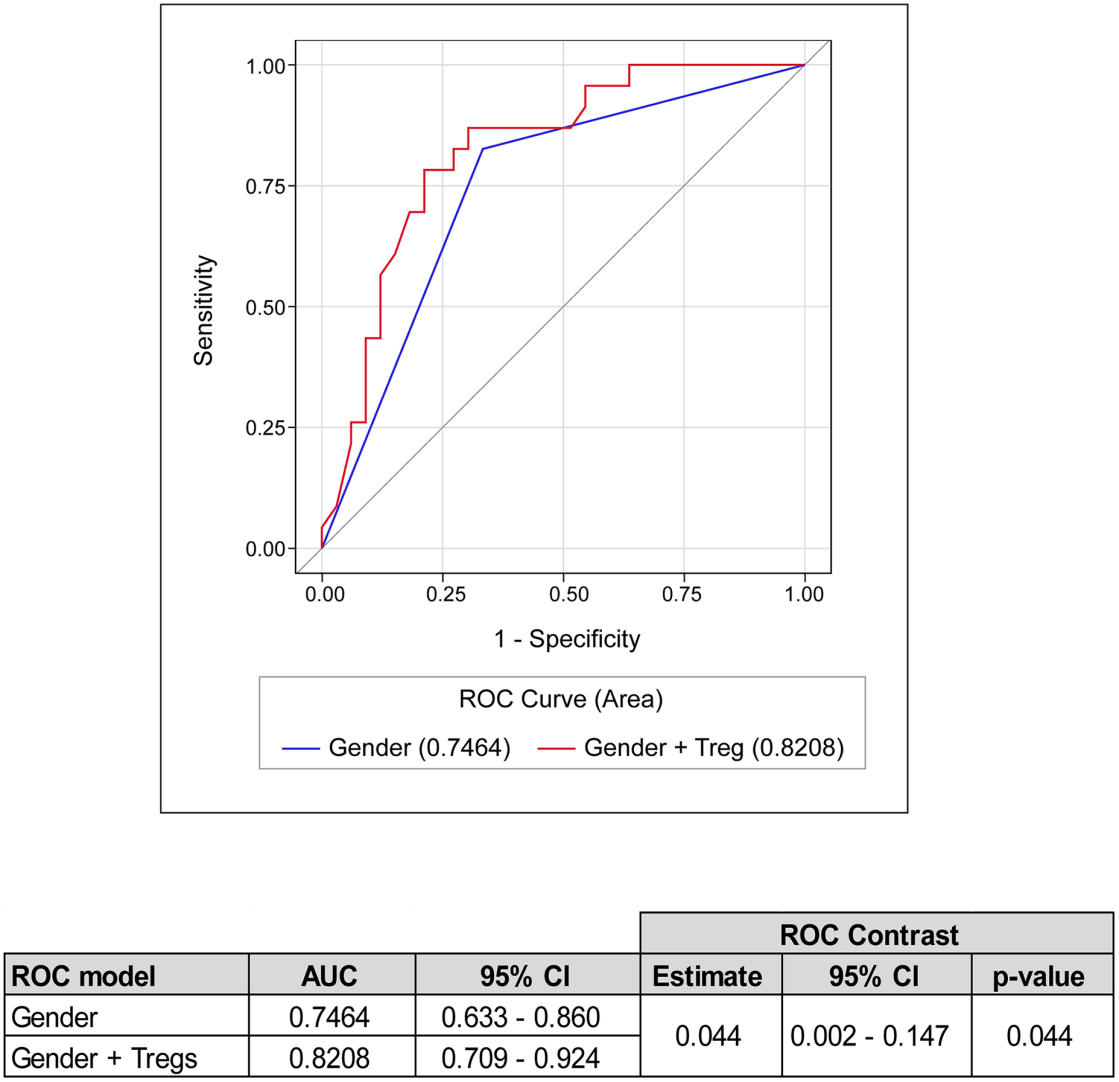
ROC curve selection analysis. ROC analysis for the discrimination of improved and worsened clinical course during hospitalisation. The best individual discriminator (i.e., gender) and the best combination (i.e., gender + Tregs) are reported. AUC, Area Under the ROC Curve; 95% CI, 95% CI confidence interval.

The multivariable logistic regression analysis revealed that subjects harbouring decreased CD4^+^, Th1, Th2 and Tregs have a significantly increased risk of worsening during the hospitalisation, after adjusting for gender, presence of comorbidity and the administration of treatment during hospitalisation (**Table 4**). In particular, patients with baseline CD4^+^ T cells ≤ 136.7 cells/µL have 6.5 times the odds of progression compared to subjects with higher count. Similarly, Th1 count ≤ 18.34 cells/µL, Th2 count ≤ 5 cells/µL and Treg ≤ 30 cells/µL are associated with increased risk of 7.9, 4.4 and 6.8 times, respectively. Similarly, patients with baseline PaO2/FiO2 ratio ≤186 have significantly higher odds of worsening. The multivariable analysis also confirmed a significantly higher risk for men to undergo clinical aggravation compared to women. The combination of gender and Tregs through a ROC curve selection analysis significantly improved the discriminatory ability of gender, with AUC increased from 0.75 to 0.82 (p=0.044) (**Figure 4**).

**Table 4:** Multivariable logistic regression analysis for the prediction of a clinical aggravation during hospitalisation.

Parameter	Odds ratio Estimates				Likelihood Ratio test			Hosmer-Lemeshow goodness of fit		
	Reference	Odds Ratio	95% CI	p-value	χ2	DF	p-value	χ2	DF	p-value
**CD4^+^ T cells**	≤ 136.7	6.471	1.513 - 27.673	0.012	21.756	5	<0.001	4.858	8	0.773
Gender	Female	0.234	0.055 - 0.989	0.048						
Age		1.042	0.984 - 1.104	0.157						
Comorbidity	No	1.822	0.272 - 12.217	0.537						
Treatment	No	1.939	0.410 - 9.160	0.403						
**Th1 (CCR6^-^/CXCR3^+^)**	≤ 18.34	7.863	1.653 - 37.396	0.010	24.204	5	<0.001	3.834	7	0.799
Gender	Female	0.156	0.031 - 0.774	0.023						
Age		1.060	0.992 - 1.132	0.085						
Comorbidity	No	3.512	0.401 - 30.799	0.257						
Treatment	No	1.776	0.369 - 8.556	0.474						
**Th2 (CCR6^-^/CCR4^+^)**	≤ 5	4.352	1.059 - 17.893	0.042	20.720	5	<0.001	3.237	7	0.862
Gender	Female	0.105	0.023 - 0.479	0.004						
Age		1.047	0.986 - 1.112	0.135						
Comorbidity	No	1.696	0.257 - 11.191	0.583						
Treatment	No	1.838	0.395 - 8.558	0.438						
**Treg (CD25^+^/CD127^low^)**	≤ 30	6.807	1.571 - 29.495	0.010	22.357	5	<0.001	4.606	8	0.799
Gender	Female	0.141	0.032 - 0.618	0.009						
Age		1.053	0.991 - 1.119	0.096						
Comorbidity	No	2.419	0.370 - 15.819	0.357						
Treatment	No	1.826	0.397 - 8.409	0.440						
**PaO2/FiO2**	≤ 186	16.678	1.925 - 144.477	0.011	24.222	5	<0.001	10.308	8	0.244
Gender	Female	0.110	0.023 - 0.529	0.006						
Age		1.032	0.974 - 1.093	0.285						
Comorbidity	No	2.064	0.308 - 13.848	0.456						
Treatment	No	1.404	0.276 - 7.138	0.683						

Treatment administered during hospitalisation included: hydroxychloroquine; corticosteroids; hydroxychloroquine + antivirals; hydroxychloroquine + corticosteroids; hydroxychloroquine + immunological treatment; hydroxychloroquine + antivirals + corticosteroids; hydroxychloroquine + antivirals + immunological treatment; hydroxychloroquine + antivirals + immunological treatment + corticosteroids.

## Discussion

Lymphopenia is a main feature of COVID-19 infection, affecting CD4^+^ and CD8^+^ T cells as well as B lymphocytes, and is more pronounced in severely ill patients (23–28). Several studies are suggesting an association between an impaired, over-activated or inappropriate T-cell response with disease severity or progression (11). In our cohort of hospitalised COVID-19 patients we showed an independent association between CD4^+^ T cell subset frequency and disease severity, with reduced CD4^+^ T cells, Th1 and Tregs showing the strongest relation with a severe clinical presentation.

Recent studies reported that SARS-CoV-2 elicits a strong and broad T cell response, both CD4^+^- and CD8^+^-mediated with, in some cases, the development of a memory phenotype, which might lead to a long-term immunity (29). However, different scenarios in the immune response to the virus have been reported and proposed to be responsible for the wide spectrum of clinical presentation of COVID-19 (30). This was also observed in our cohort, in which patients suffering from different severity of COVID-19 displayed different levels of circulating CD4^+^ T cell subsets. Systemic lymphopenia, which in our population seems to affect primarily CD4^+^ T cells, could be the consequence of cell infiltration and sequestration in the lung (10). Nonetheless, it cannot be excluded that diminished circulating lymphocytes might be associated with an impaired immune response in more severe patients, potentially associated with the presence of comorbidities. Although this latter variable did not influence cell population variability in our multivariate analysis, we could not assess the effect of specific categories of comorbidities but only their cumulative effect due to the limited sample size.

We also observed a significant association between decreased circulating CD4^+^ T cells and their subsets, but not CD8^+^ T cells, and an aggravation of patients’ clinical conditions during hospitalisation. We demonstrated that patients harbouring decreased CD4^+^, Th1, Th2 and Tregs at baseline have a significantly higher risk of clinical deterioration, independently of their gender, age, the presence of comorbidities and treatment administration. Older age and male gender have already been highlighted as important risk factors for more severe disease and clinical course (31). This association was confirmed in our population since patients’ gender was the best predictor of clinical course, when variables were considered individually (data not shown). However, the combination of gender with the number of Tregs circulating at baseline significantly improved the ability to predict clinical worsening, indicating that Treg enumeration could help in patients’ stratification according to their risk of aggravation. Compared to other cell types, Tregs have been less investigated in COVID-19, although they appear to be involved in disease progression due to their participation to innate and adaptive immune responses. In particular, in the early stage of infection they were shown to downregulate T cell-mediated immune responses, while in late stage severe COVID-19 patients they reduced the hyper-inflammation through cytokine modulation (32). In our population, reduced circulating Tregs showed a strong association with both disease severity and progression, potentially as a results of an increased recruitment to the infection site, thus lung tissues, to control the local inflammation and tissue damage. Nonetheless, we cannot exclude that this Treg reduction is associated with a functional dysregulation, as already suggested by functional analyses on bronchoalveolar lavage fluid (33).

In our study we did not observe increased circulating cytokines associated with disease severity nor with the clinical course, with the exception of IFNα that raised in worsening patients. Although the cytokine storm has been reported by many as a hallmark of COVID-19 severity and critical illness (34), our results suggest that a more complex picture might accompany disease evolution in our cohort. Systemic hyper-inflammation appears to be primarily associated with COVID-19 infection *per se* rather than with disease severity. Indeed, the majority of the studies have highlighted raised inflammatory biomarkers in infected subjects compared to healthy controls, but only few have shown differences associated with disease severity (35).

The results of our study confirm that an immune signature is associated with a more severe clinical presentation and an aggravation during hospitalisation, partly in agreement with previous observations on different patterns of immune responses elicited in hospitalised patients that might benefit from a different medical intervention based on their immune signature (3).

Our study has some limitations. The inclusion of asymptomatic subjects, in addition to our hospitalised patients, could have contributed in better determining the immunophenotype associated with COVID-19 infection, independently of disease severity. Such studies are however already available in the literature and it is important to recall that during the first COVID-19 wave in Italy asymptomatic individuals were only rarely detected. The availability of additional samples taken during hospitalisation as well as at discharge could have helped in defining a more precise picture of T cell kinetics, and particularly Treg, during the infection. Nonetheless, the comprehensive characterisation of our cohort at baseline, thanks to complete and homogeneous clinical records, has allowed achieving an in depth data analysis with the evaluation of important confounding factors including comorbidities and treatment.

In our study we did not investigate the lymphocyte activation state. Even though we could not draw any conclusions regarding the functional state of the cells of interest, we highlighted the important role of less represented whole cell populations, particularly Tregs, as potential stratification markers easily measurable in small amount of blood. In this scenario, it should be mentioned that several clinical trials evaluating strategies to improve T cell response as a therapeutic intervention for COVID-19 are currently ongoing, including T cell adoptive transfer (NCT04457726, NCT04762186, https://clinicaltrials.gov/). The potential use of T cells as diagnostic tools is also under evaluation (NCT04874818). In conclusion, we have confirmed the extensive immune-dysregulation associated with COVID-19 severity and shown the association between decreased T cell subtypes, especially of T helper lineage, and an exacerbation of patients’ clinical conditions during hospitalization. Based on their ability to predict clinical worsening, here we extend the potential utility of CD4^+^ T cells measurement, especially of Tregs, for patients’ stratification based on the risk of clinical deterioration early after hospital admission.

## Data Availability Statement

The datasets presented in this study can be found in online repositories. The names of the repository/repositories and accession number(s) can be found below: Zenodo repository at https://doi.org/10.5281/zenodo.5511828.

## Ethics Statement

The studies involving human participants were reviewed and approved by Ethical Committee of Verona and Rovigo provinces under protocol no. 63471/2020. The patients/participants provided their written informed consent to participate in this study.

## Author Contributions

CP and NT conceived the study. SC and NT designed the study. SC, MB, and MP performed the experiments. NR, AA, and PR managed sample and clinical data collection. CM and NT performed statistical analyses. SC and NT wrote the manuscript. All authors reviewed and edited the manuscript and approved the final version.

## Funding

This work was supported by the Italian Ministry of Health “Fondi Ricerca Corrente – L1P6” to IRCCS Sacro Cuore Don Calabria Hospital and by the Italian Ministry of Health - COVID-2020-12371675.

## Conflict of Interest

The authors declare that the research was conducted in the absence of any commercial or financial relationships that could be construed as a potential conflict of interest.

## Publisher’s Note

All claims expressed in this article are solely those of the authors and do not necessarily represent those of their affiliated organizations, or those of the publisher, the editors and the reviewers. Any product that may be evaluated in this article, or claim that may be made by its manufacturer, is not guaranteed or endorsed by the publisher.
