# Supplementary material for: Regulatory T Cells as Predictors of Clinical Course in Hospitalised COVID-19 Patients
Source: Front Immunol. 2021 Dec 2;12:789735. doi: 10.3389/fimmu.2021.789735 (PMC8674838; doi:10.3389/fimmu.2021.789735)
Supplement: Supplementary file 1 [file DataSheet_1.docx]

**Supplementary information**

**Regulatory T cells as predictors of clinical course in hospitalised COVID-19 patients**

Sara Caldrer^1*^, Cristina Mazzi^2^, Milena Bernardi^1^, Marco Prato^1^, Niccolò Ronzoni^1^, Paola Rodari^1^, Andrea Angheben^1^, Chiara Piubelli^1^, Natalia Tiberti^1^

*^1^ Department of Infectious – Tropical Diseases and Microbiology, IRCCS Sacro Cuore - Don Calabria Hospital, Negrar (Verona), Italy*

*^2^ Centre for Clinical Research, IRCCS Sacro Cuore - Don Calabria Hospital, Negrar (Verona), Italy*

**Table S1. Details of the antibody used for multicolour flow cytometry.**

| **Target antigen** | **Clone** | **Isotype** | **Format** | **Conc. (µg/test)*** |
| --- | --- | --- | --- | --- |
| CD45 | HI30 | Mouse IgG1, κ | PerCP-Cy^TM^5.5 | 0.25 |
| CD3 | OKT3 | Mouse IgG2a, κ | BV421 | 0.125 |
| CD4 | RPA-T4 | Mouse IgG1, κ | APC | 0.125 |
| CD8 | HIT8a | Mouse IgG1, κ | PE-Cy^TM^7 | 0.125 |
| CD19 | HIB19 | Mouse IgG1, κ | PE | 0.015 |
| CCR6 | 11A9 | Mouse IgG1, κ | BB515 | 0.5 |
| CXCR3 | 1C6 | Mouse IgG1, κ | PE-Cy^TM^7 | 1 |
| CCR4 | 1G1 | Mouse IgG1, κ | PE | 1 |
| CD25 | M-A251 | Mouse IgG1, κ | PE-Cy^TM^7 | 1 |
| CD127 | HIL-7R-M21 | Mouse IgG1, κ | PE | 0.5 |
| ** 120µL of whole blood were used for each test.*  *All antibodies were from BD Biosciences* | | |  |  |

**Table S2. Cell population absolute count and cytokine levels measured in COVID-19 patients classified according to disease severity.**

|  | **Mild (n=23)** | **Moderate (n=28)** | **Severe (n=9)** | **Kruskal-Wallis** | **Post-test p-value*^a^*** | | |
| --- | --- | --- | --- | --- | --- | --- | --- |
| **Target** | **Median (range)** | **Median (range)** | **Median (range)** | **p-value** | **Mild *vs* Moderate** | **Mild *vs* Severe** | **Moderate *vs* Severe** |
| ***Cell type [cells/µL]*** | | | | | | | |
| **Granulocytes** | 541.67 (138.33 - 1321.67) | 607.84 (91.67 - 2978.33) | 1233.33 (611.67 - 3176.67) | **0.008**** | 0.930 | **0.004**** | **0.01**** |
| **Monocytes** | 166.67 (21.67 - 421.67) | 134.50 (40 - 648.33) | 131.67 (51.67 - 351.67) | 0.677 |  |  |  |
| **Lymphocytes** | 468.33 (145 - 1843.33) | 395 (46 - 965) | 181.67 (98.33 - 448.33) | **0.001**** | 0.145 | **<0.001***** | **0.015*** |
| **CD3** | 323.33 (78.33 - 1151.67) | 245.84 (26 - 643.33) | 90 (35 - 296.67) | **<0.001***** | 0.249 | **<0.001***** | **0.006**** |
| **CD19** | 55 (3.33 - 323.33) | 34 (3.33 - 156.67) | 25 (11.67 - 100) | 0.086 |  |  |  |
| **NK** | 71.67 (18.33 - 496.67) | 73.835 (8.33 - 280) | 63.33 (16.67 - 146.67) | 0.825 |  |  |  |
| **CD4+** | 156.67 (50 - 720) | 155.84 (14 - 300) | 56.67 (10 - 116.67) | **<0.001***** | 0.692 | **<0.001***** | **0.002**** |
| **CD8+** | 93.33 (6.67 - 393.33) | 54.17 (6 - 258.33) | 21.67 (8.33 - 133.33) | **0.004**** | 0.166 | **0.0014**** | **0.039*** |
| **Th1*^b^*** | 31.67 (8.33 - 275) | 21.67 (3.33 - 118.33) | 6.67 (1.67 - 18.33) | **0.001**** | 0.691 | **<0.001***** | **0.003**** |
| **Th2*^b^*** | 10 (1.67 - 43.33) | 12.67 (1.67 - 30) | 5 (0.84 - 15) | 0.088 |  |  |  |
| **Th17*^b^*** | 56.67 (13.33 - 201.67) | 34.17 (3.33 - 138.33) | 11.67 (3.33 - 45) | **0.002**** | 0.162 | **<0.001***** | **0.025*** |
| **Treg** | 43.33 (10 - 106.67) | 30 (6.67 - 81.67) | 13.33 (1.67 - 21.67) | **0.001**** | 0.796 | **<0.001***** | **0.002**** |
| ***Cytokines [pg/mL]^c^*** | | | | | | | |
| **IFN-α** | 19.40 (9.44 - 315.20) | 22.16 (8.08 - 325.68) | 25.94 (13.28 - 260.76) | 0.926 |  |  |  |
| **IFN-γ** | 2.76 (0.02 - 524.60) | 6.80 (0.02 - 1186.76) | 1.35 (0.02 - 238.52) | 0.940 |  |  |  |
| **GM-CSF** | 1.94 (1.36 - 6.80) | 2.04 (1.20 - 33.56) | 2.14 (1.76 - 3.24) | 0.741 |  |  |  |
| **TNF-α** | 1.24 (0.01 - 36.32) | 2.04 (0.10 - 111.76) | 1.50 (0.52 - 8) | 0.797 |  |  |  |
| **IL-4** | 11.62 (11.62 - 214) | 11.62 (11.62 - 387) | 11.62 (11.62 - 23.24) | 0.351 |  |  |  |
| **IL-5** | 0.58 (0.58 - 47.04) | 0.58 (0.58 - 316.8) | 3.74 (0.58 - 18.84) | 0.523 |  |  |  |
| **IL-6** | 17.36 (0.64 - 861.52) | 26.24 (3.24 - 178.12) | 67.12 (2.76 - 142.36) | 0.133 |  |  |  |
| **IL-10** | 0.64 (0.64 - 114.72) | 0.64 (0.64 - 621.88) | 0.64 (0.64 - 120.2) | 0.436 |  |  |  |
| **IL-12p70** | 4.54 (0.06 - 325.32) | 8.24 (0.06 - 2514.16) | 14.76 (0.06 - 106.08) | 0.600 |  |  |  |
| **IL-17A** | 0.60 (0.60 - 47.44) | 0.60 (0.60 - 81.16) | 0.60 (0.60 - 6.16) | 0.176 |  |  |  |

^a^ *Dunns’ test for multiple comparisons with Bonferroni correction*

*^b^ Moderate, n=24*

*^c^ Mild, n=22; Moderate, n=27; Severe, n=8*

**Table S3. Evaluation of anti-SARS-CoV-2 IgM-S and IgG-N antibodies in the studied population.** A) Cell population absolute count in COVID-19 patients classified based on serology results, thus negative or positive for IgM-S and IgG-N. B) Correlation between cell populations and antibody indices.

| **A** | **IgM-S negative (n=18)** | **IgM-S positive (n=34)** | **p-value^b^** | **IgG-N negative (n=20)** | **IgG-N positive (n=32)** | **p-value^b^** |
| --- | --- | --- | --- | --- | --- | --- |
| **Granulocytes** | 503.33 (160 - 2438.33) | 776.67 (91.67 - 3176.67) | 0.2771 | 540 (160 - 2438.33) | 851.67 (91.67 - 3176.67) | 0.2251 |
| **Monocytes** | 132.5 (51.67 - 346.67) | 156.67 (21.67 - 648.33) | 0.4247 | 134.5 (95 - 421.67) | 156.67 (21.67 - 648.33) | 0.9925 |
| **Lymphocytes** | 401.67 (98.33 - 1843.33) | 348.33 (46 - 1496.67) | 0.1632 | 375 ( 98.33 - 1843.33) | 366.67 (46 - 1496.67) | 0.3422 |
| **CD3** | 281.67 (35 - 981.67) | 220 (86 - 1151.67) | 0.1061 | 235.84 (35 - 981.67) | 234.17 (26 - 1151.67) | 0.4575 |
| **CD19** | 28.34 (3.33 - 323.33) | 41.67 (3.33 - 240) | 0.4024 | 30 (3.33 - 323.33) | 41.67 (3.33 - 240) | 0.4237 |
| **NK** | 74.17 (36.67 - 496.67) | 68.34 (8.33 - 146.67) | 0.2726 | 76.34 (18.33 - 496.67) | 68.64 (8.33 - 146.67) | 0.4293 |
| **CD4+** | 153.34 (10 - 558.33) | 125 (14 - 720) | 0.2255 | 140.84 (10 - 558.33) | 128.34 (14 - 720) | 0.5043 |
| **CD8+** | 75.84 (11.67 - 393.33) | 47.5 (6 - 320) | 0.0568 | 62.5 (11.67 - 393.33) | 49.17 (6 - 320) | 0.3096 |
| **Th1^a^** | 31.67 (1.67 -150) | 16.67 (3.33 - 275) | 0.0787 | 21.67 (1.67 - 150) | 18.33 (3.33 - 275) | 0.3111 |
| **Th2^a^** | 12 (0.84 - 31.67) | 8.33 (1.67 - 43.33) | 0.0530 | 10 (0.84 - 31.67) | 8.33 (1.67 - 43.33) | 0.4340 |
| **Th17^a^** | 26.67 (3.33 - 138.33) | 35 (3.33 - 201.67) | 0.7875 | 40 (3.33 - 138.33) | 30 (3.33 - 201.67) | 0.3480 |
| **Treg** | 36.5 (1.67 - 106.67) | 20 (6.67 - 70) | 0.1840 | 36.5 (1.67 - 106.67) | 22.5 (6.67 - 70) | 0.1782 |

*Values are expressed as number of cells/µl, median (range)*

*^a^ IgM-S negative n= 17; IgM-S positive n=31; IgG-N negative n=19; IgG-N positive n=29*

*^b^ Mann-Whitney U test*

| **B** | **Granulocytes** | **Monocytes** | **Lymphocytes** | **CD3** | **CD19** | **NK** | **CD4+** | **CD8+** | **Th1** | **Th2** | **Th17** | **Treg** |
| --- | --- | --- | --- | --- | --- | --- | --- | --- | --- | --- | --- | --- |
| **IgM-S index** | 0.2020 | 0.2783 | 0.1070 | 0.0270 | 0.0656 | 0.2380 | 0.0514 | -0.1272 | -0.0957 | -0.0250 | -0.1557 | 0.0050 |
|  | 0.2519 | 0.1110 | 0.5471 | 0.8793 | 0.7124 | 0.1752 | 0.7727 | 0.4733 | 0.6086 | 0.8936 | 0.4030 | 0.9777 |
| **IgG-N index** | 0.0854 | -0.0108 | -0.0510 | -0.1468 | 0.0191 | 0.3054 | -0.1109 | -0.1624 | -0.1591 | -0.0708 | -0.1787 | -0.2002 |
|  | 0.6421 | 0.9531 | 0.7818 | 0.4226 | 0.9175 | 0.0892 | 0.5456 | 0.3744 | 0.4098 | 0.7153 | 0.3535 | 0.2720 |

*Correlations were computed only for patients positive for either IgM-S (n=34) or IgG-N (n=32) considering the respective indices.*

*IgM-S and IgG-N indices were computed as sample relative light unit (RLU)/ calibrator RLU.*

*Top numbers represent Spearman rho coefficient, bottom numbers represent p-value.*

**Table S4. Correlation between cell populations and systemic cytokine concentration.**

|  | **Granulocytes** | **Monocytes** | **Lymphocytes** | **CD3** | **CD19** | **NK** | **CD4+** | **CD8+** | **Th1** | **Th2** | **Th17** | **Treg** |
| --- | --- | --- | --- | --- | --- | --- | --- | --- | --- | --- | --- | --- |
| **IFN-α** | **-0.3062** | **-0.3286** | -0.1863 | -0.2007 | -0.1954 | -0.0900 | -0.1814 | -0.0850 | -0.2173 | -0.0914 | -0.2380 | -0.2043 |
|  | **0.0205** | **0.0126** | 0.1653 | 0.1345 | 0.1451 | 0.5054 | 0.1769 | 0.5298 | 0.1145 | 0.5112 | 0.0832 | 0.1274 |
| **IFN-γ** | -0.1558 | -0.0256 | -0.1522 | -0.1313 | -0.0067 | -0.0949 | -0.0461 | -0.1119 | -0.0541 | -0.0562 | -0.1663 | -0.0042 |
|  | 0.2470 | 0.8498 | 0.2583 | 0.3303 | 0.9606 | 0.4824 | 0.7332 | 0.4074 | 0.6977 | 0.6864 | 0.2294 | 0.9751 |
| **CM-CSF** | -0.2053 | -0.0453 | -0.1999 | -0.1973 | -0.0916 | -0.0863 | -0.1847 | -0.1017 | -0.2269 | -0.0788 | -0.2625 | -0.1503 |
|  | 0.1255 | 0.7381 | 0.1360 | 0.1413 | 0.4978 | 0.5232 | 0.1691 | 0.4514 | 0.0989 | 0.5713 | 0.0551 | 0.2645 |
| **TNF-α** | -0.1786 | -0.0816 | -0.2041 | -0.1974 | -0.0167 | -0.0968 | -0.1704 | -0.1585 | -0.1862 | -0.0907 | **-0.2734** | -0.0997 |
|  | 0.1839 | 0.5461 | 0.1278 | 0.1410 | 0.9021 | 0.4736 | 0.2050 | 0.2389 | 0.1775 | 0.5143 | **0.0455** | 0.4606 |
| **IL-4** | -0.0659 | -0.0248 | -0.1172 | -0.0933 | -0.0408 | -0.0345 | -0.0150 | -0.1122 | -0.0466 | -0.0454 | -0.1582 | 0.0207 |
|  | 0.6262 | 0.8547 | 0.3852 | 0.4900 | 0.7630 | 0.7992 | 0.9118 | 0.4061 | 0.7380 | 0.7445 | 0.2533 | 0.8782 |
| **IL-5** | -0.0553 | 0.0192 | -0.1869 | -0.1643 | -0.0398 | -0.0675 | -0.1025 | -0.1309 | -0.1912 | -0.0797 | -0.1968 | -0.0479 |
|  | 0.6829 | 0.8874 | 0.1640 | 0.2220 | 0.7691 | 0.6180 | 0.4482 | 0.3316 | 0.1660 | 0.5668 | 0.1538 | 0.7236 |
| **IL-6** | 0.2347 | -0.1392 | **-0.3885** | **-0.4313** | -0.2147 | -0.0937 | **-0.5417** | **-0.2764** | **-0.4820** | **-0.3259** | **-0.4966** | **-0.4959** |
|  | 0.0788 | 0.3018 | **0.0028** | **0.0008** | 0.1088 | 0.4882 | **0.0000** | **0.0374** | **0.0002** | **0.0162** | **0.0001** | **0.0001** |
| **IL-10** | -0.0459 | -0.0703 | -0.2094 | -0.2000 | -0.1253 | -0.0451 | -0.1847 | -0.1594 | -0.2539 | -0.1741 | **-0.3227** | -0.1697 |
|  | 0.7344 | 0.6032 | 0.1179 | 0.1359 | 0.3529 | 0.7389 | 0.1689 | 0.2362 | 0.0640 | 0.2081 | **0.0173** | 0.2070 |
| **IL-2** | -0.0612 | 0.0536 | -0.1301 | -0.1443 | -0.0698 | 0.0505 | -0.0738 | -0.1125 | -0.1828 | -0.0129 | -0.1572 | -0.0166 |
|  | 0.6511 | 0.6919 | 0.3347 | 0.2841 | 0.6061 | 0.7090 | 0.5852 | 0.4049 | 0.1857 | 0.9263 | 0.2563 | 0.9025 |
| **IL-17A** | -0.1551 | -0.1539 | -0.0557 | -0.0360 | -0.0221 | -0.0358 | -0.0282 | -0.0391 | -0.0860 | -0.0113 | -0.1263 | 0.0610 |
|  | 0.2492 | 0.2530 | 0.6805 | 0.7905 | 0.8704 | 0.7915 | 0.8352 | 0.7726 | 0.5363 | 0.9352 | 0.3626 | 0.6522 |

*Top numbers represent Spearman rho coefficient, bottom numbers represent p-value. Significant correlations are reported in bold.*

**Table S5. Cell population absolute count, cytokine levels and biochemical parameters measured in COVID-19 patients classified according to the clinical course during hospitalization.**

|  | **Improved (n=37)** | **Worsened (n=23)** | **Mann-Whitney U test** |
| --- | --- | --- | --- |
| **Target** | **Median (range)** | **Median (range)** | **p-value** |
| ***Cell type [cells/µL]*** | | | |
| **Granulocytes** | 753.33 (93.33 - 2978.33) | 611.67 (91.67 - 9176.67) | 0.988 |
| **Monocytes** | 170 (21.67 - 648.33) | 131.67 (40 - 351.67) | 0.284 |
| **Lymphocytes** | 448.33 (46 - 1843.33) | 303.33 (71.67 - 965) | **0.010*** |
| **CD3** | 313.33 (26 - 1151.67) | 183.33 (35 - 650) | **0.005**** |
| **CD19** | 55 (3.33 - 323.33) | 26 (3.33 - 100) | **0.013*** |
| **NK** | 76.67 (12 - 496.67) | 68.33 (8.33 - 280) | 0.267 |
| **CD4+** | 161.67 (14 - 720) | 80 (10 - 300) | **<0.001***** |
| **CD8+** | 63.33 (6 - 320) | 51.67 (8.33 - 393.33) | 0.128 |
| **Th1^a^** | 38 (3.33 - 275) | 13.33 (1.67 - 58.33) | **<0.001***** |
| **Th2^a^** | 12 (1.67 - 43.33) | 5 (0.84 - 20) | **0.014*** |
| **Th17^a^** | 41.67 (6.67 - 201.67) | 25 (3.33 - 138.33) | **0.008**** |
| **Treg** | 38.33 (6.67 - 106.67) | 18 (1.67 - 53.33) | **0.001**** |
| ***Cytokines [pg/mL]^b^*** | | | |
| **IFN-α** | 18.92 (8.08 - 325.68) | 61.22 (13.28 - 260.76) | **0.018*** |
| **IFN-γ** | 15.76 (0.02 - 1186.76) | 0.02 (0.02 - 261.96) | 0.323 |
| **GM-CSF** | 2 (1.2 - 33.56) | 1.96 (1.64 - 13.24) | 0.902 |
| **TNF-α** | 1.36 (0.1 - 111.76) | 1.38 (0.1 - 31.36) | 0.773 |
| **IL-4** | 11.62 (11.62 - 387) | 11.62 (11.62 - 214) | 0.089 |
| **IL-5** | 1.16 (0.58 - 316.8) | 1.09 (0.58 - 59.8) | 0.773 |
| **IL-6** | 23.48 (0.64 - 178.12) | 40.06 (2.76 - 861.52) | 0.245 |
| **IL-10** | 0.64 (0.64 - 621.88) | 0.64 (0.64 - 184.8) | 0.686 |
| **IL-12p70** | 7.8 (0.06 - 2514.16) | 7.38 (0.06 - 325.32) | 0.901 |
| **IL-17A** | 0.6 (0.6 - 81.16) | 0.6 (0.6 - 31.16) | 0.172 |
| ***Biochemical parameters*** | | | |
| **CRP [mg/L]** | 63.30 (3.38 - 327.5) | 88.57 (4.19 - 288.28) | 0.350 |
| **Ferritin [µg/L]** | 329.80 (33.90 - 1238) | 433 (1.49 - 1949) | 0.837 |
| **Craetinine [µmol/L]** | 74 (48 - 249) | 92 (61 - 221) | **0.006**** |
| **D-dimer [µg/L]** | 847 (200 - 5237) | 1353 (274 - 10012) | 0.110 |
| **ACE [U/L]** | 24.13 (2.11 - 101.2) | 25.87 (1.67 - 45.59) | 0.511 |
| **RT-qPCR (Ct)** | 26.5 (16 - 39) | 24 (12 - 33) | **0.020*** |
| **PaO_2_/FiO_2_** | 286 (171 - 482) | 225 (60 - 343) | **0.004**** |

*^a^ Improved, n=33; worsened, n=23*

*^b^ Improved, n=35; worsened, n=22*

**Table S6. ROC analysis and cut-off selection for the prediction of a worsened clinical course.**

| **Parameter** | **AUC** | **95% CI** | **Cut-Off** | **SE** | **95% CI** | **SP** | **95% CI** |
| --- | --- | --- | --- | --- | --- | --- | --- |
| Th1 (cells/μL) | 0.785 | 0.662- 0.901 | 18.34 | 0.783 | 0.614 - 0.951 | 0.758 | 0.611 - 0.904 |
| Th2 (cells/μL) | 0.694 | 0.555 - 0.832 | 5 | 0.522 | 0.318 - 0.732 | 0.818 | 0.687 - 0.949 |
| Treg (cells/μL) | 0.753 | 0.631 - 0.875 | 30 | 0.826 | 0.671 - 0.981 | 0.622 | 0.465 - 0.778 |
| CD4^+^ T cells (cells/μL) | 0.766 | 0.630 - 0.889 | 136.7 | 0.826 | 0.671 - 0.981 | 0.676 | 0.525 - 0.827 |
| PaO_2_/FiO_2_ | 0.722 | 0.581 - 0.864 | 186 | 0.435 | 0.232 - 0.637 | 0.971 | 0.916 - 1.000 |

*Only parameters included in the multivariate logistic regression analysis have been included.*

*AUC: area under the ROC curve; SE: sensitivity; SP: specificity; 95% CI: 95% confidence interval.*


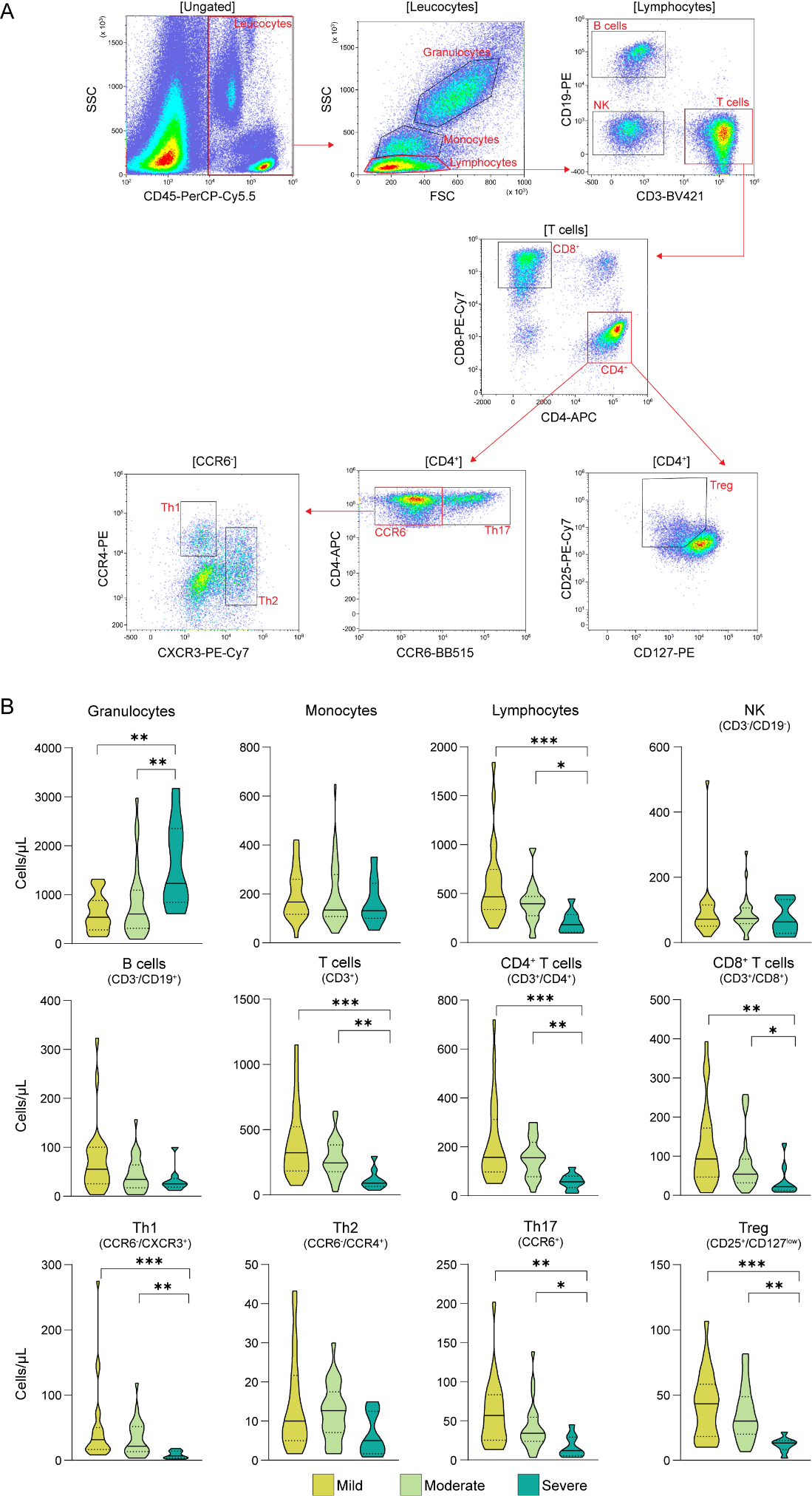


**Figure S1.** Gating strategy adopted for the enumeration of selected cell targets by multi-colour flow cytometry. Lymphocytes, monocytes and granulocytes were determined based on the FSC on the CD45^+^ leucocyte gate. B and T lymphocyte subsets were identified as CD3^+^CD19^+^ and CD3^+^CD19^-^, respectively, while NK as CD3^-^CD19^-^. T cells were then differentiated in CD4^+^ or CD8^+^. Finally, CD4^+^ T cell subsets were identified as follows: Treg (CD4^+^CD25^+^CD127^low^); Th17 (CD4^+^CCR6^+^); Th1 (CD4^+^CCR6^-^CCR4^+^); Th2 (CD4^+^CCR6^-^CXCR3^+^).


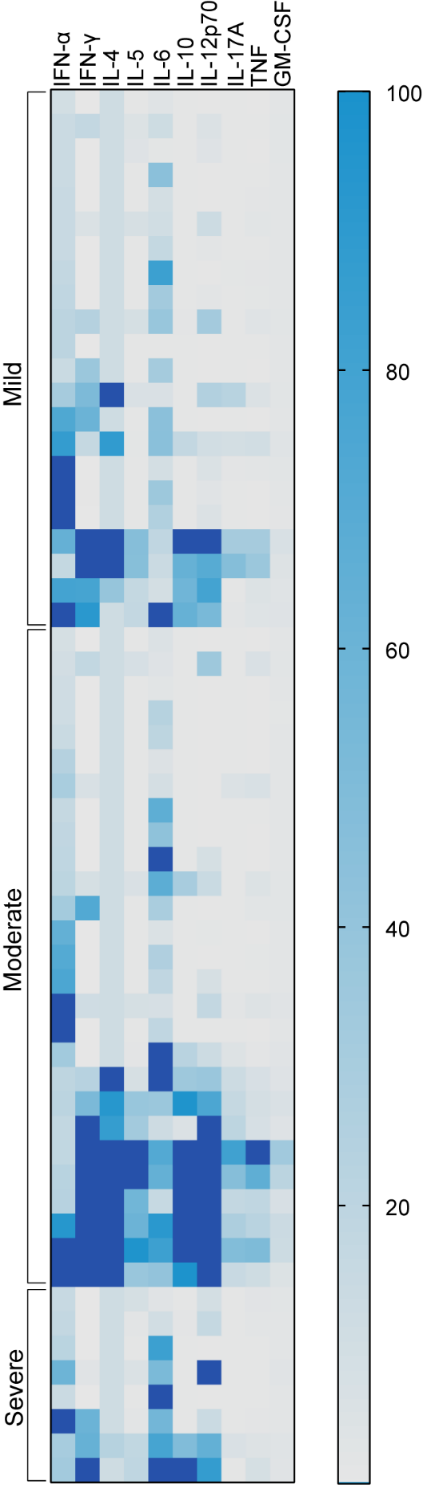


**Figure S2. Systemic cytokine profile in COVID-19 patients. A.** Heat-map showing cytokine concentration measured in COVID-19 patients. Columns represent analytes, rows represent individual subjects grouped by disease severity. Colour scale indicates cytokine concertation expressed as pg/mL. Samples having concentrations higher than 100 pg/mL are coloured in dark blue.
